# PCM Cement-Lime Mortars for Enhanced Energy Efficiency of Multilayered Building Enclosures under Different Climatic Conditions

**DOI:** 10.3390/ma13184043

**Published:** 2020-09-11

**Authors:** Cynthia Guardia, Gonzalo Barluenga, Irene Palomar

**Affiliations:** Department of Architecture, University of Alcala, 28801 Madrid, Spain; gonzalo.barluenga@uah.es (G.B.); irene.palomar@uah.es (I.P.)

**Keywords:** phase change materials (PCMs), energy efficiency, cement-lime mortar, experimental characterization, climatic conditions, heat flux

## Abstract

Phase change materials (PCMs) are promising materials for the energy efficiency improvement of building enclosures, due to their energy storage capacity. The thermal behaviour of a multi-layered building enclosure with five different compositions of PCM cement-lime mortars was evaluated under heating and cooling cycles. The behaviour of cement-lime mortars with 20% of microencapsulated PCM mixed with other additions, such as cellulose fibres and perlite, a lightweight aggregate (LWA), were studied under climate conditions of 15 °C–82% RH (cooling) and 30 °C–33% RH (heating) that were applied with a climatic chamber. Temperature and heat flux on both sides of the multi-layered enclosure were experimentally measured in laboratory tests. Temperature was also measured on both sides of the PCM cement-lime mortar layer. It was observed that the addition of the PCM cement-lime mortar layer delayed the heat flux through the enclosure. During a heating cycle, the incorporation of PCM delayed the arrival of the heat wave front by 30 min (8.1% compared to the reference mortar without PCM). The delay of the arrival of the heat wave front during the cooling cycle after adding PCM, compared to the reference mixture, reached 40.6% (130 min of delay). Furthermore, the incorporation of LWA in PCM cement-lime mortars also improved thermal insulation, further increasing energy efficiency of the building enclosure, and can be used not only for new buildings but also for energy rehabilitation of existing building enclosures.

## 1. Introduction

The design of new building materials for the energy efficiency improvement of existing buildings is a hot research topic [1,2,3]. The high energy consumption of many dwelling units built in the XX century is a main concern due to their huge carbon footprint [4,5]. Phase change materials (PCMs) are considered as a possible solution for this problem, due to their energy storage capacity [6]. During phase change, which occurs at a selected temperature that can be designed, PCMs absorb or release heat, while the material temperature remains constant (latent heat), acting as energy deposits that can be recovered when necessary [7,8]. Among the different types of PCM [7,8], the most commonly used in construction and building materials are microencapsulated paraffin waxes, which are characterized by their thermal and chemical stability without significant changes to their properties in the temperature range used in this study. They are also commercially available at a competitive price. However, paraffin waxes present low thermal conductivity and low phase change enthalpy [7,8,9,10].

Different authors have incorporated this type of microencapsulated PCM in building materials such as cement, gypsum or cement-lime mortars [8,9,10,11,12,13,14,15,16]. Besides, other additions, such as fibres (cellulose) and lightweight aggregates (LWAs, e.g., perlite), can be also incorporated to improve the thermal insulation capacity of mortars [16,17,18]. Some authors have studied the combined use of PCMs and these additions in mortars, taking advantage of both the the energy storage capacity of PCMs and the thermal insulation capacity [16]. It was found that PCM efficiency depends not only on the amount of PCM but also on the other components of the mortars [16]. The amount of PCM incorporated modified some thermal, physical and mechanical properties of mortars. Temperature range and variation also influenced the efficiency of PCM-modified mortars [15,19,20,21]. 

The experimental characterisation of materials’ properties is the first step necessary to evaluate the possibilities of their application for building purposes [1,12,13,14,16]. However, the actual effect of the newly designed materials for improving building enclosures can only be fully evaluated by considering the multi-layered composition of real enclosures. Accordingly, some authors have studied the behaviour of multi-layered specimens by assessing new mortars with enhanced properties under different climatic conditions [15,17,18,19,20,21]. Climatic chambers can be used to test specific thermal conditions, simulating real environmental conditions [15,17]. It was concluded that the PCM effect on the multilayer enclosure’s behaviour depended on the climatic conditions (summer/winter) [9,14,17,18,19,20,21,22,23,24,25].

In this paper, a study on the behaviour of a multi-layered brick wall enclosure incorporating different cement-lime mortars with the addition of 20% of PCM microencapsulated paraffin wax, LWA (expanded perlite) and cellulose fibres was carried out under different climate conditions. The aim of the study was to evaluate the thermal behaviour of the mortar layer and the overall enclosure under different climatic conditions. For this purpose, a climatic chamber was used, heating (from 15 °C to 30 °C) and cooling (from 30 °C to 15 °C) cycles were programmed and temperature and heat flux through the specimen were evaluated.

## 2. Materials and Methods

**Figure 1:**
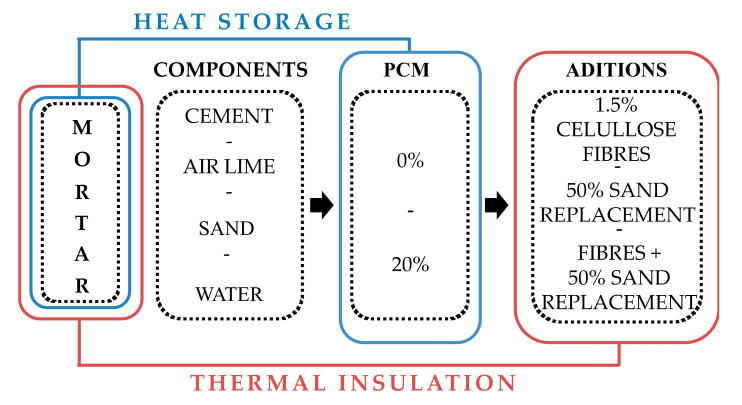
Composition design scheme of PCM cement-lime mortars.

Five PCM-modified cement-lime mortars were analyzed. Figure 1 shows the criteria followed to design mortar compositions, considering the combined effect of energy storage capacity supplied by PCMs with the enhanced thermal insulation provided by LWAs and fibres. The experimental program assessed physical and mechanical properties and thermal conductivity in both liquid and solid states of PCMs. The temperature of the inner enclosure layers and on both external sides of the wall was analyzed. Heat flux inside and outside the enclosure was also measured.

### 2.1. Material and Mortar Compositions

The components used in the study were:

The cement used was a white type cement named BL II/B-L 32.5 N (UNE-EN 197-1) from Cementos Portland Valderrivas, Madrid, Spain.

(1) CL 90-S was the air lime added to the composition, (UNE-EN 459-1).
(2) The fine aggregate was a siliceous sand with a size between 0–4 mm.
(3) The lightweight aggregate (LWA) used was an expanded perlite (L).
(4) The fibres (F) used in this study were short cellulose fibres with a length of 1 mm—Fibracel^®^ BC-1000 (Ø 20 µm) (Omya Clariana S.L, L’Arboc, Tarragona, Spain).
(5) The phase change material (PCM) chosen for the study was a microencapsulated paraffin wax (Micronal^®^ DS 5040X,) with a melting point of ca. 23 ± 1 °C supplied by BASF Construction Chemicals Company, Madrid, Spain. The bulk density of this PCM is ca. 300–400 kg/m^3^ and has a particle size which varies between 50–300 µm.

Table 1 presents the composition of the five mortars under study. Firstly, a reference cement-lime mortar (C) was produced. The cement:lime:aggregate ratio for this mortar was 1:0.5:4.5 by volume. Afterwards, 20% of PCM by volume of fresh mortar was added (C_20_). In order to improve the thermal insulation capacity of the mortar, 1.5% of dry cellulose fibres considering the total fresh mortar’s volume (regarding the reference mortar) was added (CF_20_). In addition, 50% of the sand was replaced by the lightweight aggregate (CL_20_). Finally, fibres and perlite were added (CLF_20_) in order to design a mixture of both additions. Water to binder ratio (w/b) varied in order to get for all the mixtures a similar fresh workability. The minimum compressive strength target value was 3.5 MPa, corresponding to a CS-III grade rendering mortar (medium-high strength according to UNE-EN 998-1). The mixing process in the laboratory began blending all the dry components. Then the liquid water was added. The mixing process took a maximum of 5 min.

### 2.2. Experimental Methods

#### 2.2.1. Hardened Properties and Thermal Parameters

The flow table test was used to measure the fresh mortar consistency, according to UNE-EN 1015-3:2000. In order to achieve a plastic consistency, the water to binder ratio was adjusted. Hardened state properties were characterised on 40 mm × 40 mm × 160 mm specimens, according to UNE-EN 1015-11. After 24 h, samples were demoulded and water cured for 28 days (21 ± 3 °C and 95 ± 5% RH). Bulk density (D) and open porosity (OP) (accessible to water) was calculated using a hydrostatic scale (UNE-EN 1015-10). According to UNE-EN 1015-19, the water vapour diffusion resistance factor (VD) was measured. VD was measured by the wet cup method. Cylindrical specimens with a 35 mm diameter and 40 ± 2 mm thickness with a saturated saline dissolution were used. After 28 days, compressive and flexural strength (CStr, FStr) were tested on standard specimens according to UNE-EN 1015-11:2000.

Thermal conductivity (λ) was measured using a thermally insulated box (hot box method). Plate samples with a size of 210 mm × 210 mm and a thickness of 24 ± 2 mm were used. Laboratory conditions during the test were 20 ± 1 °C and 50 ± 5% RH. A heat source was connected to a thermal regulator and located inside the insulated box. Sensors placed on the inner and outer surface of the sample and inside and outside the box monitored temperature (T) and relative humidity (RH) [26]. Two different temperatures were set inside the box: 25 °C and 40 °C. At 25 °C inside the box, the testing specimens remained below the PCM melting point (nominally 23 °C, although experimentally established at 22 °C [16]), while at 40 °C inside the box, all the plates were 30 °C. Thermal conductivities, λ_S_ (mortar’s conductivity when PCM was in a solid state) and λ_L_ (mortar’s conductivity when PCM was in a liquid state) were calculated after a thermal steady state was reached using Fourier’s Law [26]. 

#### 2.2.2. Climatic Chamber Testing Set-Up

**Figure 2:**
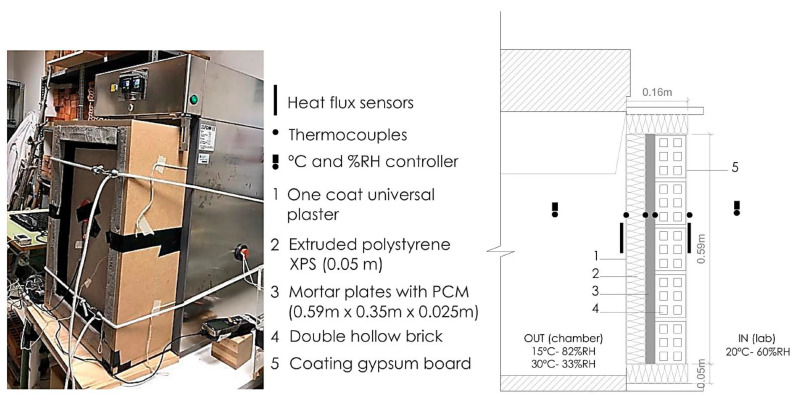
Climatic chamber set-up for monitoring the brick walls in different temperature conditions.

A climatic chamber was used to assess the thermal behaviour of brick wall enclosures with PCM cement-lime mortars under different climatic conditions. Figure 2 shows the experimental set-up used. A multi-layered hollow brick enclosure arrangement was selected [4,5], consisting of a hollow brick wall, a gypsum-based internal rendering and an external PCM-modified coating mortar covered by a 5 cm XPS (Extruded polistyrene) insulation layer (external insulation coating system, ETICS). The sample was placed inside the climatic chamber door, and the chamber conditions were modified to produce a thermal transfer through the specimen.

Two climatic conditions were tested in the chamber, simulating outdoor environmental conditions (OUT): a heating cycle and a cooling cycle. The heating cycle consisted of an initial stable condition of 15 °C and 82% relative humidity (RH), changing afterwards to 30 °C and 33% RH. The cooling cycle followed a reversed order, with an initial stable climatic condition of 30 °C and 33% RH, changing to 15 °C and 82% RH. Outside the chamber, the laboratory conditions (IN) remained constant at 20 ± 1 °C and 60% RH. RH inside the chamber was set to limit the water transport through the specimen. Each cycle (both heating and cooling) lasted 1400 min. During both cycles, heating and cooling, temperatures on both sides of the mortar layer, as well as at the external and internal sides of the enclosure, were monitored. Heat flux was also measured using a heat flux plate (Hukseflux HFP01 with an uncertainty degree of ±3%) for each side of the enclosure (Figure 2), registering the W/m^2^ on both sides with a data logger (Hobo UX120, Bourne, MA, USA). As shown in Figure 2, heat flux sensors were adhered and hermetically sealed to the surface of the material. The temperature and RH inside and outside the climatic chamber were also monitored using thermocouples, coupled temperature and RH sensors (i-button Hygrochrom^™^ DS1923 (Newbury, Berkshire, UK), with an uncertainty degree of 0.02 °C and a size of Ø 17.35 mm and a thickness of 5.9 mm, and heat flux plates, located as shown in Figure 2.

## 3. PCM Cement-Lime Mortar Physical and Mechanical Properties

Table 2 presents the experimental results obtained for workability, physical, mechanical and thermal properties of the five mortars under study. Table 2 shows that consistency values varied between 178 mm (C) and 166 mm (C_20_).

Regarding the hardened mortars’ physical properties, the reference mortar (C) presented the highest bulk density (D) value, as expected, with 1900 kg/m^3^, while the PCM mortar with LWA and fibres (CLF_20_) showed the lowest, with 1160 kg/m^3^. Open porosity (OP) values varied between 23.33% (CL_20_) and 16.77% (CF_20_). Considering the water vapour diffusion resistance factor (VD) of the mixtures under study, C_20_ showed the highest value (4.29) while CLF_20_ showed the lowest one (3.26). 

Table 2 also presents the mechanical properties of the mortars. The results for compressive strength (CStr) pointed out that C presented the highest value (14.33 MPa) while CLF_20_ showed the lowest one (5.33 MPa), which agreed with the density values. Although the 20% addition of PCM in the mixtures reduced CStr, all the mortars fulfilled the minimum compressive strength target value of 3.5 MPa, corresponding to a CS-III grade rendering mortar according to UNE-EN 998-1. On the other hand, flexural strength (FStr) values ranged between 3.36 MPa (C) and 1.79 MPa (CL_20_). 

Thermal conductivity results of the PCM mortars in both the solid state (λ_S_, T < 22 °C) and in the liquid state (λ_L_, T > 22 °C) are also summarised in Table 2. It can be observed that λ_S_ varied between 0.30 W/mK (CF_20_) and 0.20 W/mK (C_20_). Otherwise, C_20_ presented the highest value for λ_L_ with 0.28 W/mK and CLF_20_ presented the lowest one with 0.18 W/mK. 

As expected, the addition of 20% of PCM modified not only the thermal properties but also other hardened properties. Mechanical properties such as compressive and flexural strength decreased with the addition of PCM. On the other hand, λ_S_ decreased while λ_L_ increased when PCM was added, compared to the same mixture without PCM. These results were further analysed and discussed in a previous work [14].

## 4. Experimental Test Results and Thermal Analysis of Brick Wall Enclosures with PCM Mortars

The brick wall enclosures with PCM mortars were evaluated using the experimental set-up shown in Figure 2 and following the test procedure for a heating and a cooling cycle. For each sample, the thermal performance of the PCM mortar layer and the overall enclosure thermal performance were monitored during the test.

### 4.1. Thermal Behaviour of the PCM Cement-Lime Mortar Layer Inside the Enclosure Solution under Heating and Cooling Cycles

#### 4.1.1. Heating Cycle

Table 3 shows the experimental results obtained during the heating cycle on both sides of the mortar layer, measured inside the enclosure. The initial and final temperature on both sides of the layer, the outer side in contact with the XPS insulation layer and the inner side in contact with the brick wall, the average values of the mortar layer and the difference in temperature between both sides are summarised.

Initial temperatures on the outer side (XPS) of the mortar layer varied between 20.80 °C (CF_20_) and 19.00 °C (C_20_ and CL_20_), while on the inner side (B), temperatures ranged between 21.40 °C for C and 20.60 °C for C_20_ and CL_20_. Regarding final temperatures (climatic chamber conditions of 30 °C and 33% RH), CF_20_ was the mixture with the highest temperature on the XPS side (25.50 °C) and CL_20_ presented the lowest (23.40 °C). On the brick wall side, the temperature varied between 25.00 °C (CF_20_) and 21.30 °C (CL_20_). 

The average temperature of the layer can give an estimation of the solid or liquid state of PCMs. As verified in a previous study, the phase change temperature of the PCM used was 22 °C [16]. Table 3 shows that the initial average temperatures (15 °C inside the chamber) remained under 22 °C in all cases. Therefore, it can be assumed that the PCM was in a solid state. On the other hand, as the average temperature at the end of the heating cycle was above 22 °C (Table 3), it can be said that during each heating cycle, the PCM changed from solid to liquid state in all cases. 

With regard to the temperature differences between both sides of the mortar plates (XPS and brick wall), it can be observed that initially the values ranged between 1.80 °C (C) and 0.30 °C (CLF_20_), while at the end they varied between 2.10 °C (CL_20_) and 0.40 °C (CLF_20_). 

**Figure 3:**
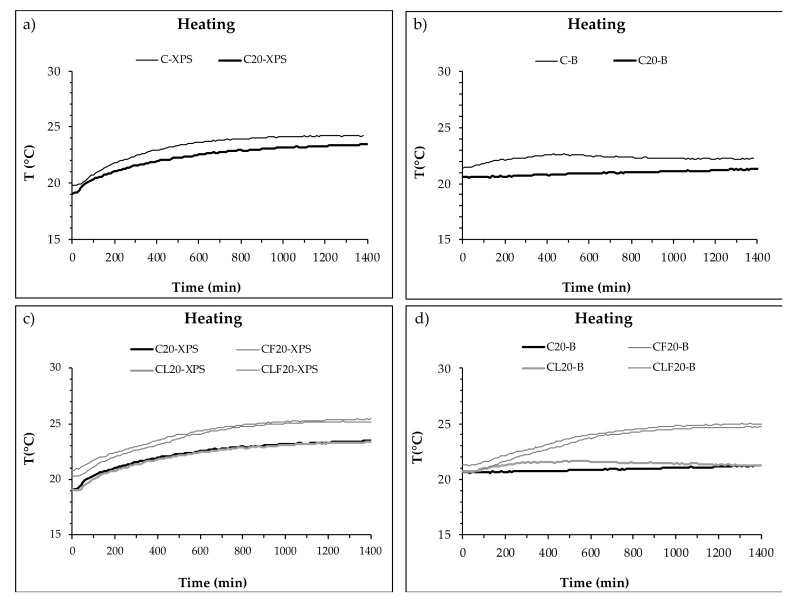
Temperature on both sides of the PCM cement-lime mortar layer during a heating cycle. (**a**) Inner side (XPS) of C and C_20_ plates. (**b**) Outer side (B) of C and C_20_ plates. (**c**) Inner side (XPS) of C_20_, CF_20_, CL_20_ and CLF_20_ plates. (**d**) Outer side (B) of C_20_, CF_20_, CL_20_ and CLF_20_ plates.

Figure 3 presents the temperature increases on both sides of the mortar layer. Figure 3a,b compare the samples with and without PCM (C and C_20_) and Figure 3c,d plot the temperature curves of samples with PCM (C_20_) and the other components (CF_20_, CL_20_ and CLF_20_).

As expected, Figure 3a shows how the temperature on the side of the enclosure in contact with the heating source (XPS) increased continuously until the stabilisation of the system (final temperature). The incorporation of 20% of PCM reduced the slope of the curve, reducing the final temperature on the surface of the mortar layer. The same effect can be observed on the inner side of the plate in contact with the brick wall (B) of the plates. Accordingly, it can be said that PCM reduces the temperature on both sides of the mortar layer. The average temperature of the layer was therefore reduced, although the difference between them was similar (Table 3). 

The enclosures with PCM mortars also showed a temperature increase due to the effect of the temperature increase inside the climatic chamber. When compared (Figure 3c,d), two groups of mixtures were identified. C_20_ and CL_20_ showed final temperatures under 23 °C and a slower increase compared to the mixtures with cellulose fibres (CF_20_ and CLF_20_), which showed temperatures over 25 °C. Temperature differences between both sides of the C_20_ and CL_20_ plates were 2 °C, while temperature differences between CF_20_ and CLF_20_ were 0.5 and 0.4 °C, respectively. These differences were more significant on the inner side of the enclosure at the end of the test. Consequently, the addition of cellulose fibres increased the overall temperature of the mortar layer.

#### 4.1.2. Cooling Cycle

Temperature was monitored on both sides of the mortar layer during the cooling cycle, which was tested after the heating cycle. Table 4 shows the experimental results obtained on both sides of the mortar layer. The initial and final temperature on both sides of the layer, the average values of the layer and the difference in temperature between both sides are summarised.

Initial temperatures on both sides, average temperature of the mortar layer and the difference in temperature were slightly different compared to the end of the previous heating cycles due to the thermal inertia of the enclosure specimen. The initial average temperature was over 22 °C for all mixtures, so PCM was in a liquid state.

Regarding the final temperature, the C mixture presented the highest value (21.50 °C) and CL_20_ the lowest one (19.70 °C) on the XPS side, while on the B side, the temperature ranged between 20.90 °C (C_20_ and CL_20_) and 21.70 °C (CF_20_). As the final average temperature was under 22 °C in all cases, PCM was in solid state after the cooling cycle. As occurred during the heating cycle, it can be stated that during the cooling cycle for each mixture, the PCM changed its phase from solid to liquid. 

Table 4 shows the values of temperature differences between both sides of the plates at the beginning and end of the cooling cycle. CL_20_ was the mixture that presented the highest value with 2.40 °C, while CF_20_ presented the lowest one with 0.40 °C. The final values ranged between 1.20 °C (CL_20_) and 0 °C (C).

**Figure 4:**
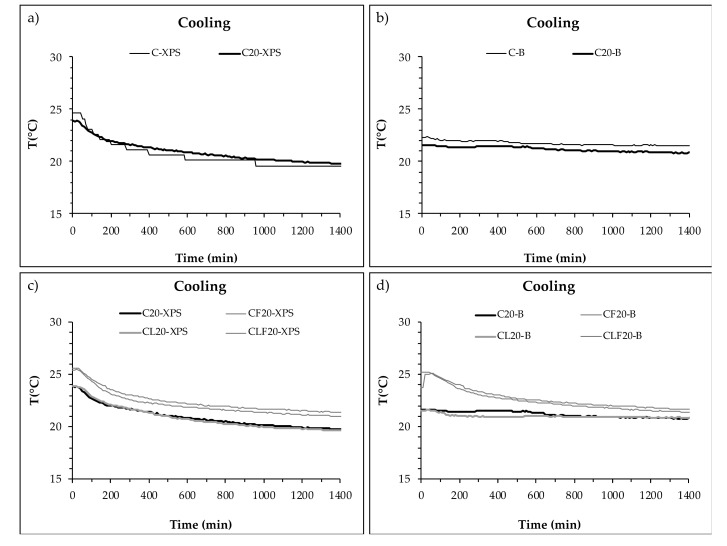
Temperature on both sides of the PCM cement-lime mortar layer during a cooling cycle. (**a**) Inner side (XPS) of C and C_20_ plates. (**b**) Outer side (B) of C and C_20_ plates. (**c**) Inner side (XPS) of C_20_, CF_20_, CL_20_ and CLF_20_ samples. (**d**) Outer side (B) of C_20_, CF_20_, CL_20_ and CLF_20_ samples.

The temperature evolution on both sides of the mortar layer, inside the enclosure, during a cooling cycle test are plotted in Figure 4: Figure 4a,b show the temperature development during cooling cycles of mortars without PCM (C) and with 20% of PCM (C_20_) and Figure 4c,d plot the temperature of mixtures with PCM (C_20_) and with the other components (CF_20_, CL_20_ and CLF_20_). 

The results obtained showed that there was no significant difference when PCM was incorporated into the mortar (Figure 4a,b). However, when cellulose fibres or LWA were added, some differences arose. Mortar with LWA only did not show significant differences in C_20_, although the use of cellulose fibres (both with or without LWA) increased the slope of the temperature curve, especially on the inner side of the enclosure (Figure 4d).

Summarising the results measured for a cooling cycle, the addition of PCM increased the temperature difference between the outer and the inner side of the mortar layer, LWA did not produce significant changes in temperature and cellulose fibres reduced the difference. C_20_ and CL_20_ showed a final temperature difference of over 1 °C, while for CF_20_ and CLF_20_ the difference was under 0.5 °C. 

For both heating and cooling cycles, mortars with LWA and 20% of PCM recorded the highest temperature differences between the outer and inner sides of the mortar layer inside the enclosure. Considering the differences between the initial and final temperatures on both sides of the mortar layer, PCM showed greater influence during the cooling cycle than during the heating cycle. On the other hand, the addition of fibres reduced the effect of PCM and PCM plus LWA on the temperature changes produced by the heating and cooling cycles.

### 4.2. Effect of the PCM Cement-Lime Mortar Layer on the Overall Thermal Performance of the Brick Wall Enclosure under Heating and Cooling Cycles

Temperature and heat flux (HF) were measured for enclosure specimens with an intermediate mortar layer with PCM, LWA and cellulose fibres. Heating and cooling cycles were applied with a climatic chamber and the results were compared.

#### 4.2.1. Heating Cycle

**Figure 5:**
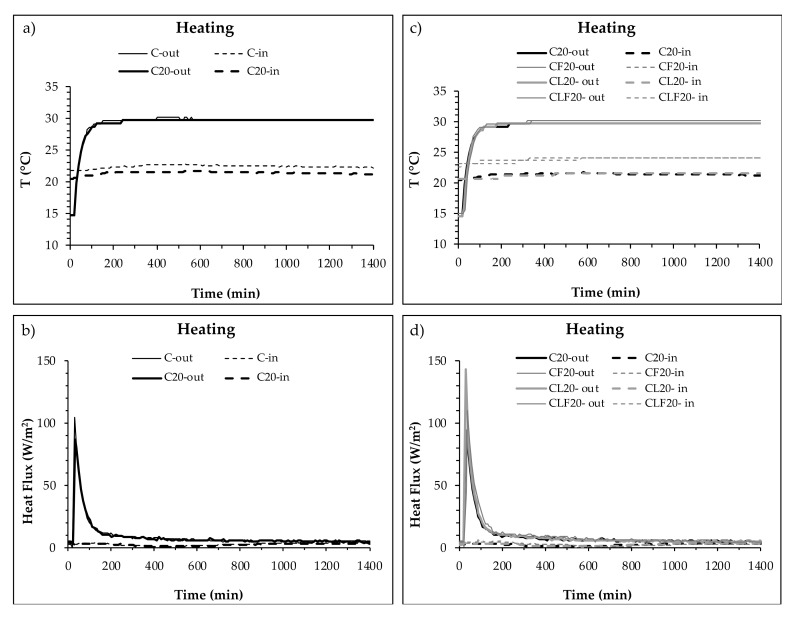
Temperature and heat flux monitored on the inner and outer sides of enclosures with a PCM cement-lime mortar layer during a heating cycle (from 15 °C to 30 °C). (**a**) Temperature of enclosures with and without PCM mortar layer (C and C_20_). (**b**) Heat flux of enclosures with and without PCM mortar layer (C and C_20_). (**c**) Temperature of enclosures with different types of PCM mortar (C_20_, CF_20_, CL_20_ and CLF_20_). (**d**) Heat flux of enclosures with different types of PCM mortar (C_20_, CF_20_, CL_20_ and CLF_20_).

The experimental results of temperature and heat flux on both sides of the enclosure during a heating cycle are plotted in Figure 5 and the main data are summarised in Table 5. Figure 5a,c relate the temperature evolution of mortars with and without PCM while Figure 5b,d compare the heat flux (HF) evolution during a heating cycle (from 15 °C to 30 °C). Figure 5a compares the temperature curve of enclosures with mortar layers without and with PCM, C and C_20_, respectively. During the test, the temperature on the external side of the enclosure increased until 30 °C was reached at 200 min, remaining constant until the end of the test. Temperatures on the inner side of the wall (in laboratory conditions) were almost constant until 200 min, increasing slightly afterwards due to the arrival of the thermal wave front. It can be observed that the addition of PCM to the cement-lime mortar layer reduced the inner temperature by 1 °C compared to the same mortar without the addition of PCM. 

Figure 5c compares the temperature of enclosures with PCM mortars with different compositions (C_20_, CF_20_, CL_20_ and CLF_20_). As expected, no difference in the outer temperature was observed. However, two groups of inner temperature curves were identified, related to whether they had cellulose fibres (CF_20_ and CLF_20_) or not (C_20_ and CL_20_). Enclosures with a PCM mortar layer with fibres reached 3 °C above the inner temperature of enclosures without fibres, which barely reached 21 °C.

The heat flux measured on both sides of the enclosures during a heating cycle is plotted in Figure 5b (mortar with and without PCM) and in Figure 5d (PCM mortars with different compositions). A general trend can be observed in the outer HF, with a sharp initial increase until a peak value, followed by a fast decrease and then a slow decrease until a final stabilisation at a steady-state heat flux.

Figure 5b,d also record the heat fluxes on the inner side of the enclosure. The curves followed a general trend, where the heat flux showed a slight decrease until the arrival of the thermal wave front and a moderate increase afterwards until reaching the steady-state stabilisation value.

Table 5 summarises temperature values on the inner side of the enclosure. The initial temperature varied between 23.11 and 20.50 °C, while the final temperature ranged between 21.20 and 24.11 °C (CF_20_ and C_20_, respectively). Enclosures with PCM mortar with cellulose fibres (CF_20_ and CLF_20_) recorded the highest values of final temperature, which can be considered the worst scenario, while C_20_ and CL_20_ showed the most advantageous behaviour. 

The peak HF values measured on the outer side of the wall (in direct contact with the climatic chamber conditions) are also presented in Table 5. The maximum peak HF value (140 W/m^2^) was measured for PCM mortar with lightweight aggregate (CL_20_) due to the lower conductivity provided by the LWA (Table 2). Accordingly, the combination of PCM and LWA showed the most advantageous behaviour, delaying the advance of the heat wave front through the enclosure.

Table 5 also presents the time of arrival of the heat wave front to the inner side of the enclosure (inner HF inflexion point). It can be observed that the enclosures with lower thermal conductivity (λ_L_ in Table 2) delayed the arrival of the heat wave to the inner side, increasing the effective thermal inertia of the enclosure.

The final HF measured after thermal and HF stabilisation showed small differences among the enclosures tested, ranging between 3.15 and 4.60 W/m^2^ on the outer side and between 1.82 and 3.15 W/m^2^ on the inner side. The small difference between the outer and the inner sides can be attributed to mass transfer related to water movement through the enclosure.

Based upon the temperature and heat flux results during the heating cycle, some analyses can be done. It can be seen that C presented an initial temperature 0.66 °C higher than C_20_, although both constructive systems were stabilised under the same climatic conditions both inside and outside. Likewise, C and C_20_ presented great differences in the temperature increase during the test, as C increased its temperature 1 °C more than C_20_, which could be related to the addition of 20% of PCM to the mortar. Regarding the heat flux measured on the inner side, the incorporation of PCM delayed by 30 min the arrival of the heat wave front, delaying the inflexion point from 370 to 400 min, which could be related to the heat storage capacity of PCM.

Considering mortars with 20% of PCM, two groups were identified related to the presence of cellulose fibres. Mixtures with fibres (CF_20_ and CLF_20_) showed an initial temperature 2–3 °C higher than mortars without fibres (C_20_ and CL_20_). This difference remained almost constant during the test. Regarding the HF peak, the compositions with lightweight aggregate showed the largest values (140 W/m^2^ and 110 W/m^2^) and, therefore, the larger delay in the heat wave front. It can be seen that the reference mixture (C) also showed similar values due to its higher density (Table 2) and, consequently, its higher thermal inertia. 

Finally, the incorporation of lightweight aggregates and cellulose fibres also produced an extra delay in the arrival of the heat wave front to the inner side of the enclosure (Table 5 and Figure 5d). Accordingly, the delay above 400 min can be attributed to the other components beyond the effect produced by PCM alone. The delay was longer for mixtures with fibres than mixtures with LWA. 

#### 4.2.2. Cooling Cycle

**Figure 6:**
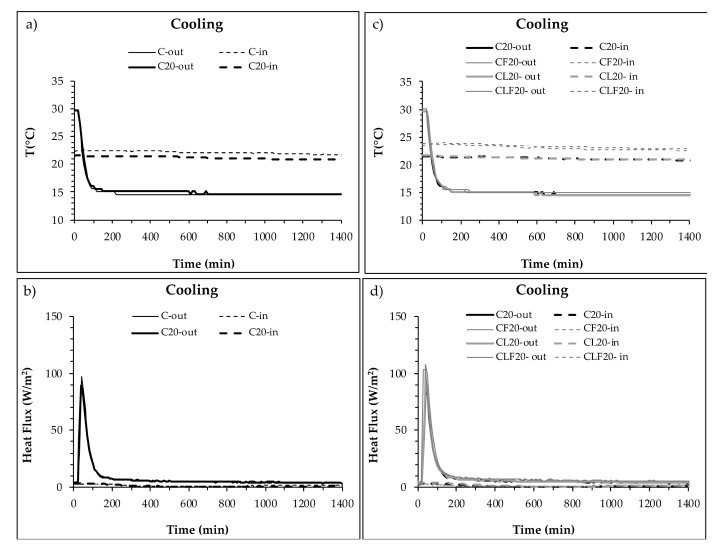
Temperature and heat flux monitored on the inner and outer sides of enclosures with a PCM cement-lime mortar layer during a cooling cycle (from 30 °C to 15 °C). (**a**) Temperature of enclosures with and without PCM mortar layers (C and C_20_). (**b**) Heat flux of enclosures with and without PCM mortar layers (C and C_20_). (**c**) Temperature of enclosures with different types of PCM mortar (C_20_, CF_20_, CL_20_ and CLF_20_). (**d**) Heat flux of enclosures with different types of PCM mortar (C_20_, CF_20_, CL_20_ and CLF_20_).

The experimental results of temperature and heat flux on both sides of the enclosure during a cooling cycle (from 30 °C to 15 °C) are plotted in Figure 6 and the main data are summarised in Table 6. Figure 6a,c relate the temperature evolution of mortars with and without PCM, while Figure 6b,d compare the heat flux (HF) evolution during a heating cycle.

Figure 6a compares the temperature curve of enclosures with mortar layers without and with PCM, C and C_20_, respectively. During the test, the temperature on the external side of the enclosure decreased until 15 °C was reached at 200 min, remaining constant until the end of the test. Temperatures on the inner side of the wall (in laboratory conditions) were almost constant until 200 min, slowly decreasing slightly afterwards due to the arrival of the thermal wave front. The addition of PCM to the cement-lime mortar layer reduced the inner temperature by 1 °C compared to the same mortar without the addition of PCM.

Figure 6c compares the temperatures of enclosures with PCM mortars with different compositions (C_20_, CF_20_, CL_20_ and CLF_20_). The temperatures measured on the outer side followed the same curve in all cases, as expected. On the inner side, as happened for the heating cycle, two groups of temperature curves can be seen, corresponding to the incorporation of cellulose fibres (CF_20_ and CLF_20_), which remained roughly 2 °C above the other mixtures. 

The heat flux measured on both sides of the enclosures during the heating cycle is plotted in Figure 6b (mortar with and without PCM) and in Figure 6d (PCM mortars with different compositions). The HF curves showed a general pattern similar to that observed for the heating cycle, although the heat moved to the opposite direction in this case. The curves of the outer HF began with a sharp initial increase until a peak value, followed by a fast decrease and a final slow decrease afterwards until a stabilisation at a steady-state heat flux.

Figure 5b,d also record the heat fluxes on the inner side of the enclosure. The curves followed a general trend, where the heat flux showed a slight decrease until the arrival of the thermal wave front and a moderate increase afterwards until reaching the stabilisation steady-state value. In the case of the cooling cycle, the addition of PCM did not cause significant changes in the thermal capacity of the mortar when it changed from liquid to solid phase. On the other hand, it can be observed that mixtures with fibres presented a higher initial temperature in comparison with the mixtures without them. This trend continued over time (Figure 6d).

Table 6 summarises temperature values on the inner side of the enclosure. The initial temperature varied between 24.11 °C (CF_20_ and CLF_20_) and 21.63 °C (CL_20_), while the final temperature ranged between 20.63 and 23.63 °C (CL_20_ and CF_20_, respectively). Enclosures with PCM mortar with cellulose fibres (CF_20_ and CLF_20_) recorded the highest values of final temperature. 

The peak HF values measured on the outer side of the wall (in direct contact with the climatic chamber conditions) are also presented in Table 6. The maximum peak HF value (110 W/m^2^) was measured for PCM mortar with cellulose fibres (CF_20_) because it also showed the highest initial temperature. However, when the time to reach the inflection point on the inner side of the enclosure, corresponding to the time of arrival of the heat wave front, was considered, CF_20_ did not show the longest delay. As happened for the heating cycle, C_20_ and CL_20_ showed the longest delays. It can be observed that the enclosures with lower thermal conductivity (λ_L_ in Table 2) delayed the arrival of the heat wave to the inner side, increasing the effective thermal inertia of the enclosure. In this case, PCM showed a greater effect (130 min) than LWA (90 min).

The final HF measured after thermal and HF stabilisation showed small differences among the enclosures tested, ranging between 3.24 and 5.13 W/m^2^ on the outer side and between 1.32 and 1.87 W/m^2^ on the inner side. The small difference between the outer and the inner sides can be attributed to mass transfer related to water movement through the enclosure.

Based upon the temperature and heat flux results during the cooling cycle, it can be seen that the inner temperature depended more on the initial inner temperature rather than on the mortar composition. Regarding the heat flux measured on the inner side, the incorporation of PCM delayed by 130 min the arrival of the heat wave front, due to the heat storage capacity of the PCM, while LWA supplied 90 extra minutes of delay. On the contrary, cellulose fibres did not increase the positive effect of the PCM.

## 5. Conclusions

An experimental study to evaluate the behaviour under different climatic conditions of a new enclosure solution containing microencapsulated phase change material cement-lime mortars with LWA and fibres inside was carried out. The experimental program assessed the thermal behaviour of the mortar layer and the overall enclosure by measuring the temperature and heat flux during heating and cooling cycles. The main conclusions of this study were:

(1) The addition of PCM to a conventional cement-lime mortar modified the physical, mechanical and thermal properties of the mortar, reducing density and strength, while increasing heat storage capacity.
(2) The addition of lightweight aggregates and fibres also modified mortar heat transfer capacity, increasing some properties already improved by PCM.
(3) The thermal behaviour of the PCM cement-lime mortars depended not only on the composition but also on the climatic conditions to which they were subjected.
(4) The addition of cellulose fibres facilitated the heat/cold transfer through the mortar layer, increasing or decreasing the average temperature of the mortars, which can be less favourable, especially in heating conditions.
(5) The addition of PCM delayed by 30 min the arrival of the heat wave front (8.1%) during the heating cycle. During the cooling cycle, the addition of PCM delayed by 130 min (40.6%) the arrival of the heat wave front compared to the reference mixture without PCM.
(6) LWA reduced thermal conductivity, increasing thermal insulation capacity and, therefore, producing an advantageous coupled effect with PCM energy storage capacity. Consequently, the combined use of PCM and LWA produced a remarkable delay of the heat wave front on the inner side of the enclosure in both heating (19%) and cooling conditions (68.7%), compared to the reference mixture.
(7) The combination of cellulose fibres and PCM showed a reduced synergic effect, but only in heating conditions.

## Figures and Tables

**Table 1:** Compositions of PCM cement-lime mortars, components in kg (adapted from [16]).

Components	C	C_20_	CF_20_	CL_20_	CLF_20_
BLII/B-L 32.5N	348	348	348	348	348
Air lime	55	55	55	55	55
Fine aggregates	1403	1403	1403	702	702
Fibres	-	-	0.66	-	0.66
LWA	-	-	-	94	94
PCM	-	84.6	84.6	84.6	84.6
Liquid water (*)	220	200	240	250	380
w/b (*)	0.73	0.68	0.78	0.71	0.79
D_dry_ (kg/m^3^)	1400	1357	1440	868	-
D_wet_ (kg/m^3^)	2264	1937	1885	1562	1561

* Fine aggregate humidity (5.3%) was also considered.

**Table 2:** Physical, mechasnical and thermal properties of the PCM cement-lime mortars (adapted from [16]).

Properties	C	C_20_	CF_20_	CL_20_	CLF_20_
Consistency (mm)	178	166	170	170	170
D (kg/m^3^)	1900	1600	1660	1270	1160
OP (%)	19.56	17.72	16.77	23.33	23.09
VD	4.13	4.29	3.47	3.62	3.26
CStr (MPa)	14.33	7.17	5.83	6.00	5.33
FStr (MPa)	3.36	2.40	2.20	1.79	2.16
λ_S_ (W/mK)	0.23	0.20	0.30	0.29	0.23
λ_L_ (W/mK)	0.21	0.28	0.23	0.18	0.15

**Table 3:** Temperatures on both sides of the PCM cement-lime mortar layer measured inside the enclosure during the heating cycle (from 15 °C to 30 °C).

Temperatures	C	C_20_	CF_20_	CL_20_	CLF_20_
Initial T_i_ (°C)					
Outer side (XPS)	19.60	19.00	20.80	19.00	20.30
Inner side (B)	21.40	20.60	21.30	20.70	20.60
Final T_f_ (°C)					
Outer side (XPS)	24.23	23.50	25.50	23.40	25.20
Inner side (B)	22.22	21.60	25.00	21.30	24.80
Layer average T_i_	20.50	19.80	21.05	19.85	20.45
Layer average T_f_	23.23	22.55	25.25	22.35	25.00
T difference T_i_	1.80	1.60	1.30	1.70	0.30
T difference T_f_	2.01	1.90	0.50	2.10	0.40

**Table 4:** Temperatures on both sides of the PCM cement-lime mortar layer measured inside the enclosure during the cooling cycle (from 30 °C to 15 °C).

Temperatures	C	C_20_	CF_20_	CL_20_	CLF_20_
Initial T_i_ (°C)					
Inner side (B)	22.30	21.60	25.20	21.50	23.80
Outer side (XPS)	24.10	23.90	25.60	23.90	25.40
Final T_f_ (°C)					
Inner side (B)	21.50	20.90	21.70	20.90	21.40
Outer side (XPS)	21.50	19.80	21.40	19.70	21.00
Layer average T_i_	23.20	22.75	25.40	22.70	24.60
Layer average T_f_	21.50	20.35	21.55	20.30	21.20
T difference T_i_	1.80	2.30	0.40	2.40	1.60
T difference T_f_	0	1.10	0.30	1.20	0.40

**Table 5:** Summary of temperature and heat flux main results on both sides of the brick wall enclosure with a PCM cement-lime mortar layer during a heating cycle.

Thermal Parameters	C	C_20_	CF_20_	CL_20_	CLF_20_
Initial inner T_i_ (°C)	21.13	20.50	23.11	20.63	22.61
Final inner T_f_ (°C)	22.63	21.20	24.11	21.63	24.11
T_inner_ difference (°C)	1.5	0.7	1	1	1.5
Max. outer HF (W/m^2^)	105	90	95	140	110
Inner infl. point (min)	370	400	580	440	540
Final outer HF (W/m^2^)	3.24	4.60	3.92	3.15	4.47
Final inner HF (W/m^2^)	1.82	3.15	3.15	2.55	2.55

**Table 6:** Summary of temperature and heat flux main results on both sides of the brick wall enclosure with PCM cement-lime mortar layer during a cooling cycle.

Thermal Parameters	C	C_20_	CF_20_	CL_20_	CLF_20_
Initial inner T_i_ (°C)	22.63	22.13	24.11	21.63	24.11
Final inner T_f_ (°C)	21.63	21.13	23.61	20.63	23.11
T_inner_ difference (°C)	1	1	0.5	1	1
Max. outer HF (W/m^2^)	100	90	110	95	105
Inner infl. point (min)	320	450	270	540	270
Final outer HF (W/m^2^)	3.24	3.24	3.92	4.47	5.13
Final inner HF (W/m^2^)	1.82	1.32	1.82	1.87	1.87

## Nomenclature

PCM	Phase change material	RH	Relative humidity
F	Cellulose fibre	T_i_	Initial temperature
LWA	Lightweight aggregate	T_f_	Final temperature
D	Bulk density	XPS	Insulation layer
OP	Open porosity	B	Brick layer
VD	Water vapour resistance factor	HF	Heat flux

## References

[1] Palomar I., Barluenga G., Puentes J. (2015). Lime–cement mortars for coating with improved thermal and acoustic performance. Constr. Build. Mater..

[2] Sala E., Zanotti C., Passoni C., Marini A. (2016). Lightweight natural lime composites for rehabilitation of Historical Heritage. Constr. Build. Mater..

[3] Bentchikou M., Guidoum A., Scrivener K., Silhadi K., Hanini S. (2012). Effect of recycled cellulose fibres on the properties of lightweight cement composite matrix. Constr. Build. Mater..

[4] Terés-Zubiaga J., Martín K., Erkoreka A., Sala J., Escudero K.M. (2013). Field assessment of thermal behaviour of social housing apartments in Bilbao, Northern Spain. Energy Build..

[5] Terés-Zubiaga J., Campos-Celador A., González-Pino I., Escudero-Revilla C. (2015). Energy and economic assessment of the envelope retrofitting in residential buildings in Northern Spain. Energy Build..

[6] Ryms M., Januszewicz K., Kazimierski P., Zaleska-Medynska A., Klugmann-Radziemska E., Lewandowski W.M. (2020). Post-Pyrolytic Carbon as a Phase Change Materials (PCMs) Carrier for Application in Building Materials. Materials.

[7] Sharma A., Tyagi V., Chen C., Buddhi D. (2009). Review on thermal energy storage with phase change materials and applications. Renew. Sustain. Energy Rev..

[8] Cabeza L.F., Castell A., Cabeza L.F., De Gracia A., Fernandez A.I. (2011). Materials used as PCM in thermal energy storage in buildings: A review. Renew. Sustain. Energy Rev..

[9] Ryms M., Klugmann-Radziemska E. (2019). Possibilities and benefits of a new method of modifying conventional building materials with phase-change materials (PCMs). Constr. Build. Mater..

[10] Rao V.V., Parameshwaran R., Ram V.V. (2018). PCM-mortar based construction materials for energy efficient buildings: A review on research trends. Energy Build..

[11] Jayalath A., Nicolas R.S., Sofi M., Shanks R., Ngo T., Aye L., Mendis P. (2016). Properties of cementitious mortar and concrete containing micro-encapsulated phase change materials. Constr. Build. Mater..

[12] Pavlík Z., Fořt J., Pavlíková M., Pokorný J., Trník A., Černý R. (2016). Modified lime-cement plasters with enhanced thermal and hygric storage capacity for moderation of interior climate. Energy Build..

[13] Lucas S.S., Ferreira V., Aguiar B. (2013). Latent heat storage in PCM containing mortars—Study of microstructural modifications. Energy Build..

[14] Cunha S., Lima M., Aguiar B. (2016). Influence of adding phase change materials on the physical and mechanical properties of cement mortars. Constr. Build. Mater..

[15] Mankel C., Caggiano A., Ukrainczyk N., Koenders E. (2019). Thermal energy storage characterization of cement-based systems containing microencapsulated-PCMs. Constr. Build. Mater..

[16] Guardia C., Barluenga G., Palomar I., Diarce G. (2019). Thermal enhanced cement-lime mortars with phase change materials (PCM), lightweight aggregate and cellulose fibers. Constr. Build. Mater..

[17] Palomar I., Barluenga G., Ball R.J., Lawrence M. (2019). Laboratory characterization of brick walls rendered with a pervious lime-cement mortar. J. Build. Eng..

[18] Wi S., Yang S., Park J.H., Chang S.J., Kim S. (2020). Climatic cycling assessment of red clay/perlite and vermiculite composite PCM for improving thermal inertia in buildings. Build. Environ..

[19] Alonso C., Oteiza I., García-Navarro J., Martín-Consuegra F. (2016). Energy consumption to cool and heat experimental modules for the energy refurbishment of façades. Three case studies in Madrid. Energy Build..

[20] Arıcı M., Bilgin F., Nižetić S., Karabay H. (2020). PCM integrated to external building walls: An optimization study on maximum activation of latent heat. Appl. Therm. Eng..

[21] Rathore P.K.S., Shukla S.K. (2020). An experimental evaluation of thermal behavior of the building envelope using macroencapsulated PCM for energy savings. Renew. Energy.

[22] Fachinotti V., Bre F., Mankel C., Koenders E.A.B., Caggiano A. (2020). Optimization of Multilayered Walls for Building Envelopes Including PCM-Based Composites. Materials.

[23] Kishore R.A., Bianchi M.V., Booten C., Vidal J., Jackson R. (2020). Optimizing PCM-Integrated Walls for Potential Energy Savings in U.S. Buildings. Energy Build..

[24] Qiao Y., Yang L., Bao J., Yang L., Liu J. (2019). Reduced-scale experiments on the thermal performance of phase change material wallboard in different climate conditions. Build. Environ..

[25] Khan R.J., Bhuiyan Z.H., Ahmed D.H. (2020). Investigation of heat transfer of a building wall in the presence of phase change material (PCM). Energy Built Environ..

[26] Herrero S., Mayor P., Olivares F.H. (2013). Influence of proportion and particle size gradation of rubber from end-of-life tires on mechanical, thermal and acoustic properties of plaster–rubber mortars. Mater. Des..

